# Prediction of caregiver quality of life in amyotrophic lateral sclerosis using explainable machine learning

**DOI:** 10.1038/s41598-021-91632-2

**Published:** 2021-06-10

**Authors:** Anna Markella Antoniadi, Miriam Galvin, Mark Heverin, Orla Hardiman, Catherine Mooney

**Affiliations:** 1 School of Computer Science, University College Dublin, Dublin 4, Ireland; 2 FutureNeuro, SFI Research Centre for Chronic and Rare Neurological Diseases, Royal College of Surgeons in Ireland, Dublin 2, Ireland; 3 Academic Unit of Neurology, Trinity Biomedical Sciences Institute, Trinity College Dublin, Dublin 2, Ireland; 4 Department of Neurology, Beaumont Hospital, Dublin 9, Ireland

**Keywords:** Motor neuron disease, Quality of life, Machine learning, Predictive medicine

## Abstract

Amyotrophic Lateral Sclerosis (ALS) is a rare neurodegenerative, fatal and currently incurable disease. People with ALS need support from informal caregivers due to the motor and cognitive decline caused by the disease. This study aims to identify caregivers whose quality of life (QoL) may be impacted as a result of caring for a person with ALS. In this study, we worked towards the identification of the predictors of a caregiver’s QoL in addition to the development of a model for clinical use to alert clinicians when a caregiver is at risk of experiencing low QoL. The data were collected through the Irish ALS Registry and via interviews on several topics with 90 patient and caregiver pairs at three time-points. The McGill QoL questionnaire was used to assess caregiver QoL—the MQoL Single Item Score measures the overall QoL and was selected as the outcome of interest in this work. The caregiver’s existential QoL and burden, as well as the patient’s depression and employment before the onset of symptoms were the features that had the highest impact in predicting caregiver quality of life. A small subset of features that could be easy to collect was used to develop a second model to use it in a clinical setting. The most predictive features for that model were the weekly caregiving duties, age and health of the caregiver, as well as the patient’s physical functioning and age of onset.

## Introduction

Amyotrophic Lateral Sclerosis (ALS), also known as Motor Neuron Disease (MND) is a rare neurodegenerative disease, with an incidence rate of 2–3 per 100,000 individuals per year. It is characterised by the progressive death of upper and lower motor neurons, causing muscle atrophy and paralysis, and it has a fatal trajectory usually within 3–4 years from symptom onset^1^. Due to the lack of one specific test to identify ALS, diagnosis can be a rather long process of medical examinations to rule out the presence of other conditions, that could last approximately a year^2^. This process alone can be exhausting for people with ALS (PALS) and their family.

It is important for patients to have support from informal caregivers, such as family members or close friends. However, these new duties associated with caregiving, as well as the condition of their loved ones may have a great impact on the caregivers’ quality of life (QoL). QoL is defined as the general well-being of a person, and it includes the individual’s perception of their physical, social, and psychological state^3^. It has previously been observed that both the patient and next of kin are affected by ALS in terms of their QoL^4,5^, and some studies have found the psychological distress experienced by caregivers to have been even higher than that of PALS^6,7^. Knowledge of the factors that are associated with the caregivers’ QoL can lead to more tailored support for them. The types of support could be financial, psychological, education relating to the condition, or related to the patient’s care and supports (e.g. equipment, therapists, access to services, respite care)^8^.

The aim of this study is to examine a variety of features and discern their link to caregiver QoL which will inform the provision of the necessary supports. To date, literature on this topic is limited and the investigation approach has been the use of descriptive statistics, multivariate and bivariate analyses. One of the studies has found caregiver age to be one of the factors associated with QoL; more specifically, younger caregivers demonstrated worse QoL^9^. Moreover, PALS’ neurobehavioral symptoms have been shown to significantly relate to lower caregiver QoL^10^. A study in India found that caregiver burden and QoL had a negative correlation^11^. In Ireland, QoL of caregivers of PALS was associated with caregiver burden, anxiety and depression^12^, but in a study of spousal ALS caregivers, family, hobbies and social activities were reported as the main contributors to improved QoL^7^. Fatigue has been referred to as a symptom that was present for both PALS and caregivers, but the small number of available treatments and the uncertainty that surrounds their efficacy are currently a challenge^6^. In our previous work we identified features that are predictive of QoL in PALS^13^, and we used a small subset of these factors in the development of an explainable model to alert clinicians when a patient is at risk of low QoL^14^. Additionally, we have developed a model for the prediction of burden in the carers of PALS, using both information from the caregiver and the PALS^15^, and using patient information exclusively^16^. We found that the patient’s age at disease onset, ALS Functional Rating Scale Revised (ALSFRS-R) score for orthopnoea and the caregiver’s status pre-caregiving were sufficient to predict patient QoL with good accuracy, while the weekly caregiving duties, age, and health of the caregiver, as well as the patient’s physical functioning and age of onset were highly predictive of caregiver burden.

Our work will add to this group of studies by introducing a different perspective in terms of the methodology. With the usage of machine learning techniques, we aim to provide insight into more complex interactions between features in order to gain additional information on the predictors of caregiver QoL, or to confirm existing findings. The limited number of studies and study participants due to the rarity of the condition creates the need for further investigation. In the current work we have a rich dataset available, consisting of a variety of features to assist in capturing different elements of QoL, as this is, by definition, a multidimensional outcome. A Clinical Decision Support System (CDSS) is one way of making health care professionals aware of a caregiver’s QoL at the point of care. CDSS can be embedded in the patient’s Electronic Health Records, so as to incorporate the information that is stored there, and provide alerts or suggest a decision. In this work, the purpose of the CDSS is to augment a provider’s perspective when meeting a patient and accelerate the necessary actions for the support of both patient and caregiver. The goal is to accomplish this without increasing the clinician’s workload, as it will be an automatic process that will require input of only a small amount of information.

To summarise, in this work we are working towards: 1) identifying predictors of the QoL of caregivers of PALS, and 2) creating a usable and explainable predictive model of caregiver QoL that can subsequently lead to the development of a CDSS to be used in clinical practice. This latter model could be incorporated in a system that will combine our previous work to alert clinicians on patient QoL as well as caregiver QoL and burden. This way healthcare decision-makers can have a more holistic view of the impact of the disease and devise an appropriate individualised support plan.

## Methods

### Study participants and data collection

Ethical approval for this study was granted by the Beaumont Hospital Research Ethics Committee under reference number REC Ref. Number: 12/84 on 07/12/2012. All research was performed in accordance with relevant guidelines and regulations.

Data were collected from interviews with 90 PALS and their primary informal caregiver. The primary caregiver was identified as such by the patient (usually the person who provided the majority of care in the form of unpaid support). Participants were interviewed at home at three time-points (at four-month to six-month intervals) between May 2013 and June 2015. The patients attended the National ALS/MND multidisciplinary clinic (MDC) at Beaumont Hospital, Dublin. All participants were identified through the MDC and provided informed written consent for the interviews and for access to the patients’ clinical information through the National ALS/MND Register. All information was pseudonymised after collection and before conducting any analysis for this work.

Each patient-caregiver pair’s interview was treated as a separate instance in our analysis. The questionnaires included demographic and socioeconomic questions, resource use, QoL, and anxiety and depression questionnaires, as well as a questionnaire of burden evaluation specific to the caregiver. Finally, information on the patients’ diagnosis, their cognitive and behavioural status, and information retrieved from the clinic visit form was collected through the National ALS/MND Register. Demographic information on the PALS and caregivers in this cohort has been previously presented in our study on caregiver burden in ALS^15^. For the patients’ disease state both the Milano-Torino (MiToS) functional staging^17^ and King’s clinical staging^18^ systems were used and included in the analysis. The El Escorial^19^ criteria were used to determine diagnosis, while the ALSFRS-R^20^ was used to measure disease progression. Each individual functionality is rated between 0 and 4, where 4 shows normal functionality. The total score ranges between 0 and 48. To assess the patients’ cognitive and behavioural impairment, the Edinburgh Cognitive and Behavioural ALS Screen (ECAS)^21,22^ and Beaumont Behavioural Inventory (BBI)^23^ were used. Symptoms of anxiety and depression were measured with the Hospital Anxiety and Depression Scale (HADS)^24^, and caregiver burden was calculated based on the Zarit Burden Interview (ZBI)^25^. High scores in HADS or ZBI are equivalent to presence of anxiety, depression or burden respectively, while low scores indicate no issue. We only included the total Anxiety and the total Depression scores from the HADS questionnaire, and the total burden score from the ZBI questionnaire (namely ZBI).

**Figure 1:**
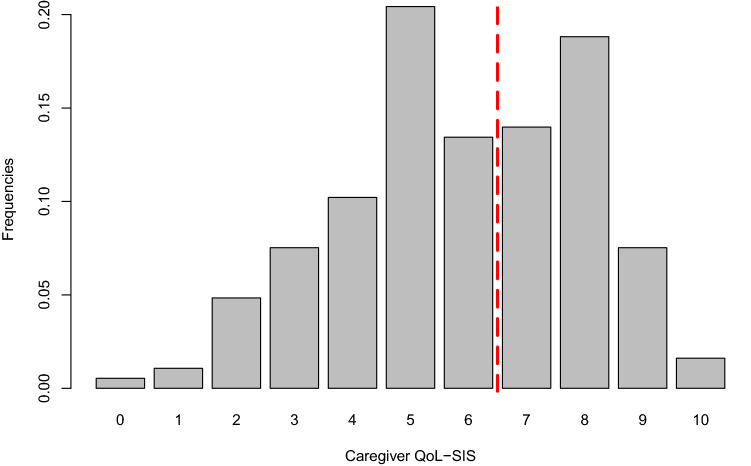
Barplot of MQoL-SIS scores. The vertical line between 6 and 7 demonstrates the way the data was split. The value 6 was the median of the distribution and was included in the low QoL class.

QoL was measured with the use of the McGill Quality of Life questionnaire (MQoL)^26^. Each question ranges from 0 (“very bad”) to 10 (“excellent”). There are five QoL sub-measures calculated from the questions in the McGill Questionnaire: Physical Symptoms; Physical Well-being; Psychological; Existential; and Support. These sub-scores were used as predictors in the analyses while the individual questions were excluded. Additionally, there is a Single Item Score (SIS) that measures the overall QoL as it is assessed by the respondent. The total QoL can also be measured by adding the five sub-measures’ scores, but MQoL-SIS is selected as the outcome of interest in this work to ease validation of our methodology and results; a single item is easier to collect than administering the full questionnaire. The distribution of MQoL-SIS scores is shown in the barplot in Fig. 1. MQoL-SIS was split in two classes according to the median value (equal to 6) to create a binary outcome.

A higher than median value is equivalent to high quality of life and is represented by 0 in our data, while a lower than median value is equivalent to a low quality of life and is represented by 1 (shown in Eq. 1).

1 $$\begin{aligned} Binary\; QoL= \left\{ \begin{array}{ll} 1\; or\; low \; &{} QoL \; score\ \le median\; value, \\ 0\; or\; high\; &{} QoL \; score\ > median\; value. \\ \end{array} \right. \end{aligned}$$

### Data pre-processing

In order to prepare the data for analysis, the first step was to remove the instances that had a missing value in the outcome, MQoL-SIS. Subsequently, some of the qualitative features were either transformed to numeric values or excluded from the analysis. More specifically, the occupation, name of organisation the respondent worked for, and business employer, as well as information about who paid for each of the aids or appliances the patient used, the hospital admission information (apart from the total number of admissions) that included time, duration and reason for admission were omitted due to their complexity. However, information related to the respondent’s occupation or in-patient admissions was included in other forms: a variable that included the reasons for admittance in the Accident and Emergency Department (A&E) was transformed into a binary feature of whether the reason was related to a fall or not (fall, removing stitches from fall, broken arm, broken leg). The remaining reasons appeared one or two times only, so they were omitted. Moreover, we created a binary feature on whether there was a change in employment status due to MND (0=no, 1=yes), instead of having the reason in text. Categorical variables that described employment status and principal economic status were available and were included as well. Regarding the features we transformed for usability purposes: four binary variables were created to represent the site of onset (Bulbar, Spinal, Cognitive, Respiratory). If there were multiple sites of onset, these were represented with the use of the value 1 in the feature that was present and 0 in the one that was absent. Similarly, we created four binary variables for the first symptom: speech, swallow, upper limb, lower limb. Only those with respiratory onset had respiratory symptoms, and only those with cognitive onset had cognitive symptoms, so no variable was created for those. The open-ended questions on drug usage were turned into three different binary features with the most common drugs or category of drugs: “Rilutek”, “Amytriptyline”, Anti-spasmodic drugs. Each questionnaire about QoL, burden, anxiety and depression was summarised in total scores, so this also reduced the number of features in the data. Due to concerns of introducing bias to the data, features with more than 30% of missing information were also omitted. The final dataset consisted of 186 rows (out of 190 before the pre-processing step), each row representing a PALS-caregiver pair at a specific time-point, and 210 predictive features (columns excluding the outcome) which is approximately 50% of the raw dataset. This will be referred to as the “Full” dataset, which was used for the development of the first (“Full”) model for the identification of the predictors of QoL. QoL has many aspects so we used a variety of features in order to approach its prediction as best we can.

For our second aim, we then used a subset of 73 features, which are either routinely collected at the point of care or accessible, either as a once-off piece of information, or through connected electronic information, to create a model that could be potentially used in a CDSS in a clinical setting. As a result, we have manually excluded all financial information such as allowances or expenditures and medications due to concept drifts, and information that requires the administration of questionnaires such as the Zarit Burden Interview, and Hospital Anxiety and Depression scores. Finally, as a step to reduce the number of features, we included only the summaries of aids and appliances i.e. the number of walking, posture, and home aids and appliances, rather than each individual item usage (an additional 24 features). The dataset for this part of the study included patient and caregiver demographic information, caregiver duties, and patient use of services and clinical information. We used this subset of features to create the first model for CDSS development, which will be referred to as the “Full-CDSS” model in this paper. A more detailed description of the selected features is presented in the list below:

1. Demographics, caregiving duties and use of services:
  Hours of care per week provided by the primary caregiver, caregiver age, health condition, respite care offer.
  Patient’s primary medical certificate, medical card ownership and condition, palliative services use, activity status before symptoms, visits to and from the healthcare professionals.
  Patient and caregiver sex, age, number of children, housing status, car access, marital status, current and pre-ALS employment status, current principal economic status, education, health insurance coverage.
2. Patient’s clinical information:
  Age and site of onset, first symptom (lower or upper limb), El Escorial diagnostic criteria, Cognitive and Behavioral Impairment scores (ECAS, BBI)
  Visit to the A&E department due to fall
  ALSFRS-R individual scores, cognitive and behavioural impairment, MITOS and King’s stage
  Number of walking, posture, and home aids and appliances used.

In both our sub-studies, the “Full” model is followed by “simpler” models using only a subset of the features that had the biggest impact on the predicted outcome.

### Data analysis

In order to create the predictive models, we first needed to set aside a proportion of the data to use for testing purposes only, in this case 25%, selected at random. The remainder 75% of the dataset was used to train the algorithm. The split in training and test sets was done in a random manner, using the method train_test_split from sklearn.model_selection in Python, with a random state equal to 123. Seventy-nine caregivers belonged to the low QoL class, and 60 belonged to the high QoL class in the training set, with a total of 139 instances. In the test set of 47 instances, 29 were in the low QoL class and 18 in the high QoL class. We used the Least Absolute Shrinkage and Selection Operator(LASSO)^27^ to create a baseline logistic regression model for comparison. The model was developed in R version 4.0-2 using the the “glmnet” function, and to determine the lambda parameter for it we used the cv.glmnet method from the “glmnet”^28^ package. Missingness is an issue in those techniques, so we performed a missing data imputation using the missForest^29^ imputation method in R.

Extreme Gradient Boosting (XGBoost)^30^ is a popular implementation of the gradient tree boosting method^31^, which was used for the development of the predictive models in this work. This technique creates an ensemble of decision trees by gradually evaluating the predictions they make, and assigning a higher importance, in the form of a weight, to the misclassified instances. This way, in the next model, those instances have a higher priority and an opportunity to be classified correctly. No imputation method was applied in this part of the study as XGBoost automatically handles missing data; it learns the appropriate branch directions for missing values during training^30^. There are several hyperparameters available for the algorithm’s tuning, and for the identification of the most appropriate ones for each model we performed an exhaustive search over different values in five-fold cross-validation. “GridSearchCV” from the sklearn package was used for the search, with the F1 score (scoring=“f1”) as its evaluation metric. The explored and the selected (best) values for each hyperparameter in this exhaustive search are shown in Tables 1 and 2 respectively. “Eta” refers to the step shrinkage to prevent overfitting, “gamma” to the minimum loss reduction required to make a new split in a tree, “min_child_weight” is the minimum sum of weights required in a child, “subsample” is the subsample of samples to be selected for the creation of each tree, “colsample_bytree’ refers ot the subsample ratio of columns to create each tree, and “alpha” and “lambda” are the L1 and L2 regularization terms on weights. Moreover, the number of decision trees in the ensemble was set to 1000, and the maximum depth of each tree in the ensemble was set to 4, while the default value of 0 was used for the seed to account for randomness. This process was repeated for both sets of models that aimed to address the two research questions in this work: 1) the identification of features related to caregiver QoL, and 2) the development of predictive models for the creation of a CDSS to automatically predict caregiver QoL.

**Table 1:** Hyperparameter values used in the grid search for the tuning of the XGBoost models.

Hyperarameter	Set of values
eta	[0.001, 0.01, 0.1, 0.2, 0.3]
gamma	[0.05, 0.5, 1, 1.5]
min_child_weight	[5, 7, 9, 10]
subsample	[0.5, 0.8, 1]
colsample_bytree	[0.6, 0.8, 1]
lambda	[0.1, 0.5, 1]
alpha	[0, 1, 2]

**Table 2:** Hyperparameter tuning of predictive models.

Model	**Hyperpameters**						
	Colsample_bytree	Eta	Gamma	Lambda	Min_child_weight	Subsample	Alpha
Baseline				0.4867818			1
Full	0.6	0.1	0.5	1	5	0.5	2
M7	0.6	0.1	0.05	0.5	7	0.8	1
Baseline-CDSS				0.039492			1
Full-CDSS	0.8	0.3	0.05	0.5	10	0.8	0
M10-CDSS	0.8	0.1	0.05	0.5	5	0.8	2
M6-CDSS	0.6	0.3	1	1	5	1	2

The Baseline models used LASSO regression while the rest used the XGBoost. For the latter, the number of estimators was 1000 and the maximum depth of each tree was set to 4.

Finally, for the models’ evaluation we selected a number of different metrics which were used to evaluate the performance on both the training and test sets. Those metrics included the recall, precision, F1 score and Area Under the Receiver Operator Characteristic Curve (AUC-ROC Curve). The ROC curve is a graph that plots the True Positive Rate (TPR) against the False Positive Rate (FPR) and the AUC-ROC measures the area under it. For better visualisation of the precision and recall we also present the Precision-Recall curves. The formulas are presented below:

$$\begin{aligned} Recall= & {} \frac{TP}{P} \\ Precision= & {} \frac{P}{TP+FP} \\ F1= & {} 2 \times \frac{Precision \times Recall}{Precision + Recall}, \\ TPR= & {} \frac{TP}{TP+FN} \\ FPR= & {} \frac{FP}{FP+TN} \end{aligned}$$

where P stands for Positives, TP for True Positives, FP for False Positives, FN for False Negatives, and TN for True Negatives.

### Explainable AI and analysis of misclassifications

In this work it was important to provide explanations regarding the features that were the most predictive of the outcome. Our baseline model used a technique that is explainable by design, but for the remaining models we required *post-hoc* explanations. For the provision of *post-hoc* explanations we used SHAP (SHapley Additive exPlanations)^32^, which is a unified approach for measuring feature importance. This methodology not only provided us with global explanations regarding the most important features, but allowed for the reduction of the feature space and the development of additional models with a smaller number of features (the most important ones). We used Tree Explainer^33^ which provides local and global explanations and interactions in tree-based models, such as the XGBoost.

Moreover, to add to the transparency of the CDSS models, we subsequently analysed the misclassified instances. The Mann–Whitney U test was used to compare the median values between the numeric features in the best CDSS model. More specifically, we tested the hypothesis that the medians of the two groups were different. For the categorical and ordinal features’ comparison, a Chi-squared test was used to compare distributions rather than median values. So in this case we tested the hypothesis that the distributions of values between the groups were different. Our aim was to test the null hypothesis that caregivers belonging in the same QoL class (e.g. 1) had significant differences among them when they were correctly and incorrectly classified, i.e., we compared caregivers that were correctly classified as “1” to those incorrectly classified as “0”. We repeated this process for all features of the opposite class (correct “0” with incorrect “1”). The level of significance used was 0.05.

## Results and discussion

QoL is an important outcome in chronic diseases for people with the disease and those providing care. In ALS in particular, caregivers are key figures in the care of patients and should receive support in order to cope with the challenges that the disease poses. In this study our first goal was the creation of knowledge regarding the factors that are related to the QoL of caregivers in ALS. The second goal focused on the development of a model to predict caregiver QoL in a usable and explainable manner, towards the future construction of a CDSS. It has been proposed that routine clinical evaluation of individual QoL make communication of PALS and caregivers with clinicians easier and it can prove informative for care-planning^7^. In this study we worked towards automating and accelerating this process. The outcome of interest was MQoL SIS as it is a single question and, as opposed to issuing the entire questionnaire to measure total QoL, it makes the future evaluation of our findings and models easier and faster.

### Identification of predictors of caregiver QoL

We first worked towards uncovering the predictors of caregiver QoL. For this task we developed predictive models which included all the information that was available in this study, excluding features with significant missingness. The baseline model was created with the use of LASSO regression as a way to automatically reduce the feature space. We then used the XGBoost algorithm which was selected due to its ease-of-use and performance across many domains, leading to many wins in machine learning competitions^30^. Performance of the baseline model was close to that of the XGBoost models, but recall was significantly lower. Because XGBoost is an algorithm that is considered black-box, we applied a post-hoc explainability method, namely SHAP, to gain insight into the features that were most predictive of the outcome. SHAP is a state-of-the-art eXplainable Artificial Intelligence (XAI) technique that offers the opportunity to extract both local explanations, for one specific prediction, and global explanations for an overview of the model’s structure.

**Figure 2:**
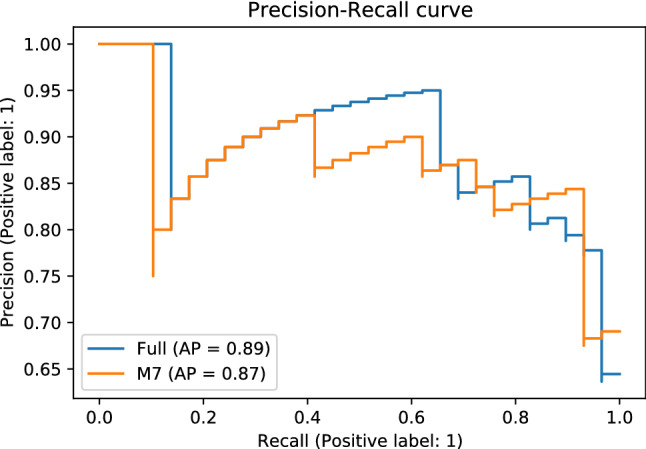
Precision-Recall Curves for the predictive models that were developed to identify the predictors of QoL.

**Figure 3:**
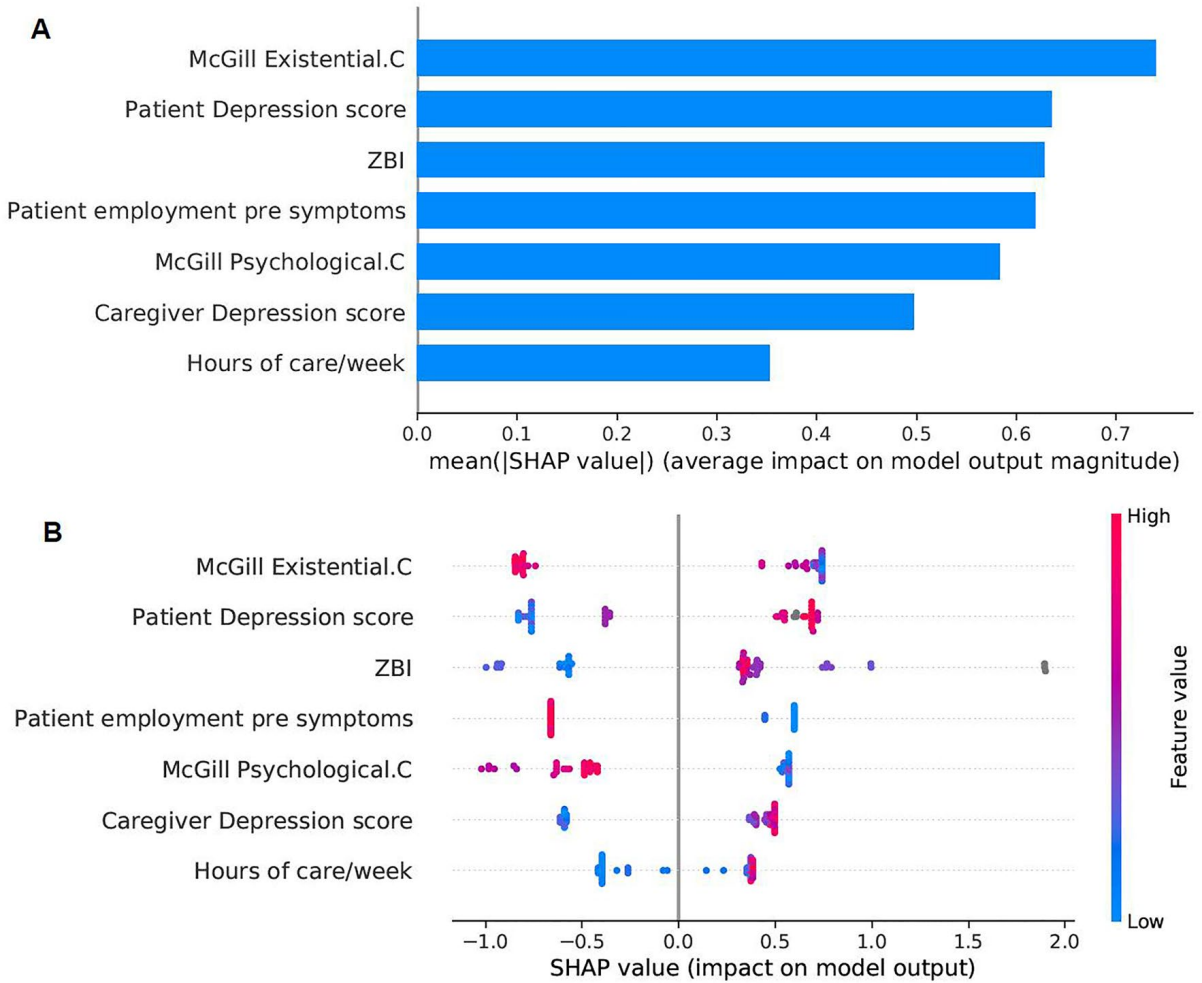
Bar plot and summary plot of model M7 for the identification of predictors of caregiver QoL. (**A**) Bar plot showing features in order of importance based on the mean absolute value of the SHAP values for each feature. (**B**) Summary plot where features appear in order of their sum of SHAP value magnitudes, and SHAP values show the impact each feature has on the model output. The colour represents the feature value (red high, blue low). The suffix “.C” represents a caregiver characteristic.

With the global explanations provided by SHAP, we visualised the overall impact that features had on the predicted outcome and then we selected a subset of the seven most predictive ones to develop a simpler model (M7). Simpler models have the potential to be more understandable, less impacted from some possibly noisy or irrelevant features, more accurate, and more usable. The selected subset of features is sufficient to predict the outcome almost with the same power as the full dataset (Table 3). The Precision-Recall curves are presented in and Fig. 2. The bar plot in Fig. 3 shows those features in order of importance by summarising all the individual predictions to provide an overall picture of their impact; this is based on the mean absolute value of the SHAP values of each feature. The variable with the highest impact on the outcome is the caregiver’s existential QoL sub-score. The next three features with similar importance among them are the patient’s depression score, caregiver burden (total ZBI score) and the patient’s employment status before symptom onset. The caregiver’s psychological QoL sub-score is the next most important feature, followed by the caregiver’s depression score and the caregiver’s hours of caregiving duties per week. The patient’s pre-symptom employment status is a categorical feature where: 1 refers to paid employment, 2 to self-employed, 3 to unemployed, 4 to student or on training course, 6 to retired, and 7 to Permanent sickness or disability. Although the performance of the baseline model was lower, it is worth mentioning that the features that contributed the most to the prediction in this model, based on the coefficient value they were assigned by the LASSO method, were similar to the ones previously described in this paragraph. In addition to those, caregiver marital status, and respiratory site of onset, bath lift ownership and medical card ownership of the patient were also found related to caregiver QoL according to the baseline model. It is important to emphasise here that correlation does not necessarily imply causation.

**Table 3:** Evaluation of predictive models for the predictors of QoL.

Model	Training				Test			
	f1	Recall	Precision	AUC	f1	Recall	Precision	AUC
Baseline	0.86	0.78	0.94	0.86	0.76	0.72	0.81	0.72
Full	0.91	0.94	0.89	0.89	0.84	0.83	0.86	0.80
**M7**	**0.87**	**0.89**	**0.86**	**0.85**	**0.83**	**0.83**	**0.83**	**0.77**

The “Baseline” and “Full” models use the entire dataset, while “M7” uses the seven most important features that resulted from the “Full” model. The best model is presented in bold. AUC is the Area Under the ROC (Receiver Operator Characteristic) Curve.

The impact that the explored features had on the predicted outcome can be seen in the summary plot in Fig. 3. In the summary plots, each dot represents a different instance in the dataset and the colour represents the feature value (red is high, blue is low)^33^. All feature values on the left of the vertical line at 0 have a negative impact on the predicted outcome while those on the right have a positive impact. If there is a high concentration of the same colour on one side of the graph, then we can see that the feature represented by that colour are generally contributing towards a specific prediction (low or high). In Fig. 3, low existential or psychological QoL sub-scale scores for the caregiver were associated with lower QoL and vice versa, but there are middle values of the predictor that were related to either one of the classes. Depression of the patient or caregiver, or high caregiver burden are indicators of lower caregiver QoL. We also found that retirement or permanent sickness/disability of a PALS before symptom onset related to higher caregiver QoL, while pre-symptom paid employment related to lower caregiver QoL. Finally, a large number of caregiving hours per week was associated with low caregiver QoL, but fewer hours could be associated with either high or low QoL.

These findings are in accordance with previous studies that have found a relationship between the caregiver’s QoL and sense of burden and depression^12^. Weekly caregiving duties and caregiver depression that were associated with QoL in this study, have been previously found predictive of caregiver burden in the same cohort^15^. Existential well-being has not been identified as a predictor of caregiver QoL in ALS, but it had been found to be significant for the QoL of PALS^34^. Interestingly, in this study, the patient’s employment status before symptom onset is predictive of the caregiver’s QoL, while in our previous study, the caregiver’s employment status before the caregiving duties onset was predictive of patient QoL^14^.

The current findings can be combined with existing research on caregiver QoL in ALS to make suggestions for the supports that can be provided. A recent study in Ireland proposed that the MDC should have the necessary staff to help caregivers maintain their well-being and patients address any psychosocial issues, as part of their visits to the clinic^7^. Education of both patients and caregivers regarding care, equipment, emotional or financial supports is a form of intervention mentioned in literature^35–37^, that could prove useful and impact the features that this work found to relate with caregiver QoL. Based on that, occupational and physical therapy services and advice to improve endurance and muscle strength could be beneficial for both the PALS and caregiver, as they could have an effect on caregiving hours.

### Modelling caregiver QoL for clinical usage

**Figure 4:**
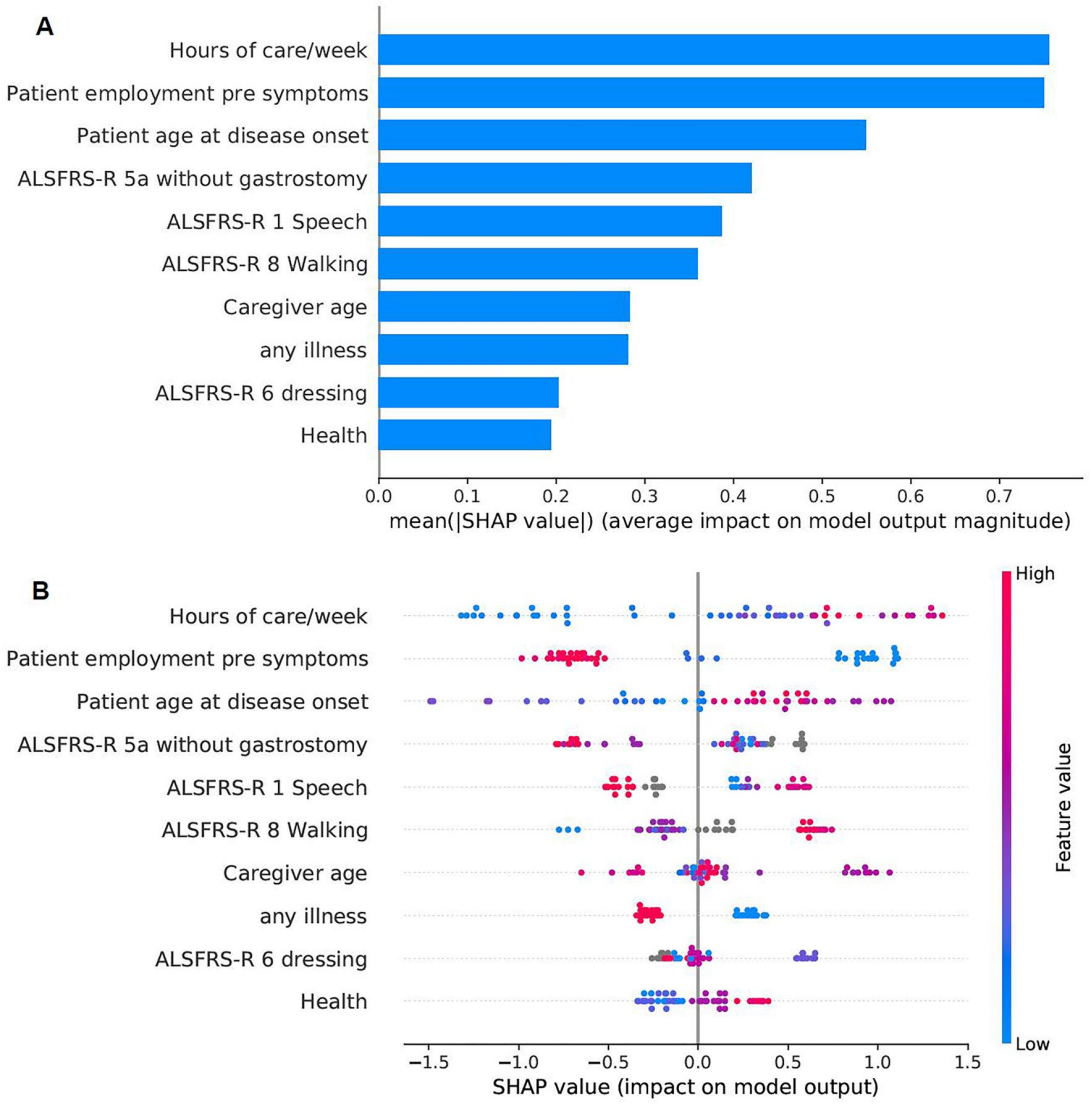
Bar plot and summary plot of model “M10-CDSS”. (**A**) Bar plot showing features in order of importance based on the mean absolute value of the SHAP values for each feature. (**B**) Summary plot where features appear in order of their sum of SHAP value magnitudes, and SHAP values show the impact each feature has on the model output. The colour represents the feature value (red high, blue low).

**Figure 5:**
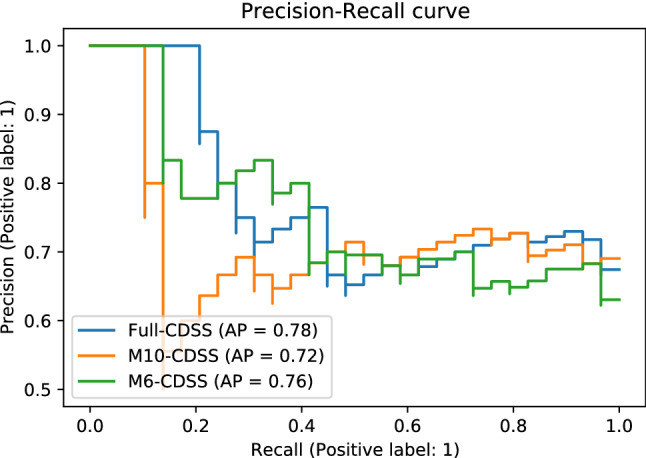
Precision-Recall Curves of CDSS models.

For our second aim, we selected a subset of features that would be readily available or that would be possible to collect at the point of care in order to develop a model for the future construction of a CDSS. We wanted a model that would use a small set of easy-to-collect features to make predictions in order to increase its usability. We applied the same process and methods that we applied for the first task. With the help of SHAP we selected smaller subsets with the most important features in the dataset in order to create a simpler model. Additionally, we used the SHAP techniques for global and local explainability to gain insight to the decision-making process of our model, that can also be presented to the clinicians. In our previous work towards a CDSS to provide alerts on patient QoL, we have demonstrated how local explanations using SHAP can explain a specific decision, with the help of some exemplar case studies^14^. The ten most important features were selected first, and they improved the predictive power. Afterwards, we tried creating a model with six of those features, but the performance was reduced, so the best model for this problem was the 10-feature model (“M10-CDSS”). According to the global explanations (Fig. 4), the features with the highest impact on the predicted outcome were the weekly hours of caregiving duties as well as the patient’s employment status before symptom onset. Important to the predictions is also the patient’s age at disease onset, the caregiver’s age, and the caregiver’s health status. The latter is expressed by two variables: “any illness” is binary and describes whether the caregiver has any long-term illness, health problems or disability (1, yes/2, no), and the “Health” feature answers the question “In general, would you say your health is:” and ranges between 1 (Excellent) to 5 (Poor). Finally, the patient’s functionality in different areas measured by the ALSFRS-R scale was also predictive of the caregiver’s QoL. These areas included speech, walking, dressing and the patient’s ability to cut food when they haven’t undergone a gastrostomy (ALSFRS-R 5a). These findings are in agreement with our previous work on modelling caregiver burden for the creation of a CDSS, where we observed similar predictors for caregiver burden, as well as a reduction in the predictive power compared to the model that used the entire spectrum of information available (“Full” model)^15^. The patient’s age of onset was also identified as the most predictive feature of patient QoL^14^, which shows a connection between the two outcomes. The evaluation metrics that were compared are presented in Table 4 and Fig. 5. We can see that the models overfit the training dataset as the performance is reduced on the test sets.

**Table 4:** Evaluation of the CDSS models.

Model	Training				Test			
	f1	Recall	Precision	AUC	f1	Recall	Precision	AUC
Baseline-CDSS	0.71	0.59	0.87	0.73	0.52	0.45	0.62	0.50
Full-CDSS	0.87	0.89	0.86	0.85	0.71	0.72	0.70	0.61
**M10-CDSS**	**0.91**	**0.92**	**0.90**	**0.90**	**0.75**	**0.79**	**0.72**	**0.65**
M6-CDSS	0.82	0.87	0.77	0.76	0.70	0.72	0.68	0.58

The “Baseline-CDSS” and “Full-CDSS” models use the whole dataset, while the numbers next to the remaining model names indicate the number of features they use that were selected based on importance. The best model is presented in bold. AUC is the Area Under the ROC (Receiver Operator Characteristic) Curve.

In terms of the type of association the predictors have with the outcome, we see that caregiving hours have a positive impact on the predicted outcome, which means that more caregiving hours are associated with lower QoL, which could be expected. The patient’s employment status before symptom onset has a similar relationship with the outcome as in the first task, where retirement and permanent sickness/disability were connected to higher caregiver QoL and paid employment was connected to lower QoL. Regarding the patient’s age of onset, a later onset is related to a lower QoL score for the caregiver. A patient’s ability to cut food when they haven’t undergone a gastrostomy, is associated with better caregiver QoL; speech functionality has a similar effect. Normal walking ability, however, has the opposite impact on the predicted QoL, as it is associated with lower scores. The presence of any long-term illness, health problems or disability on the caregiver is related to lower QoL, and their overall self-assessed health score shows the same effect. These findings suggest the need for frequent evaluation of the caregiver’s health status and the provision of support. Indeed the lives of caregivers of PALS can be stressful and they often spend most of their time caring for their loved ones and less time for themselves^38^.

#### Analysis of misclassifications

As part of the work towards a model for clinical usage, we also analysed the misclassified data, while we usually only evaluate the model in terms of the correctly classified instances. We are presenting information about the incorrectly predicted outcomes in each class in terms of their quantity and their difference to the correctly predicted ones. We performed statistical tests in order to compare the feature values between the correct and the incorrect classifications. So, we compared the True Positives (TP) with the False Negatives (FN) to discover if there was a difference between the two that lead to the misclassification of the latter in the proposed CDSS model. Similarly we compared the True Negatives (TN) with the False Positives (FP). For numeric features we used a Mann–Whitney U test and the outcomes are shown in Table 5 with their median values (M) and the tests’ *p* values. For categorical or ordinal features we used a Chi-squared test and Table 6 shows the results of the comparisons in the form of *p* values. The values in bold show statistically significant differences between the two groups, while for the rest we could not reject the null hypothesis.

**Table 5:** Mann–Whitney U test results for comparisons between TP (True Positives or Correct 1) and FN (False Negatives or Incorrect 0), as well as TN (True Negatives or Correct 0) and FP (False Positives or Incorrect 0) of numerical features in the proposed CDSS model.

Feature	TP	FN	*p* Value	TN	FP	*p* Value
Hours of care/week	M=44	M=29.5	**0.036**	M=14	M=50	**0.002**
Caregiver age	M=57	M=62.55	0.394	M=58.9	M=51.5	0.205
Patient age at disease onset	M=62	M=61.2	0.311	M=59.9	M=54.9	**0.041**

We present the median values (M) that are compared, followed by the *p* value of the test. The values in bold show statistically significant differences between the two groups, while for the rest we could not reject the null hypothesis.

To summarise Table 5, the caregiving hours per week were significantly different between the correctly and incorrectly classified values of both classes. For the patient’s age of onset the only significant difference was between the correctly and incorrectly classified negatives (TN and FP), while for the caregiver’s age we didn’t find any significant differences. Regarding the results in Table 6, we see a statistically significant difference in the distribution of values in both tests for ALSFRS-R 5a and ALSFRS-R 6, while no difference was found in ALSFRS-R 8. A significant difference between only the correctly and incorrectly classified positives (TP and FN) was noted in ALSFRS-R 1 and (caregiver’s) Health. The opposite result is found for the binary feature “Any Illness” and the patient employment status before symptom onset.

**Table 6:** Chi-squared test results for comparisons between TP (True Positives or Correct 1) and FN (False Negatives or Incorrect 0), as well as TN (True Negatives or Correct 0) and FP (False Positives or Incorrect 0) of categorical and ordinal features in the proposed CDSS model.

Feature	TP and FN *p* value	TN and FP *p* value
Any illness	0.066	**0.039**
Health	**0.011**	0.800
Patient employment pre symptoms	0.055	**0.01**
ALSFRS-R 6 Dressing	**0.017**	**0.038**
ALSFRS-R 5a Without Gastrostomy	**0.003**	**0.045**
ALSFRS-R 8 Walking	0.239	0.12
ALSFRS-R 1 Speech	**0.006**	0.14

In the table we present the *p* value of the comparison of their distributions. The values in bold show statistically significant differences between the two groups, while for the rest we could not reject the null hypothesis.

We conclude that there is a variety of differences in the features that lead to the misclassification of caregivers by the CDSS model, with the caregiver duties, and PALS functionality in cutting food without having undergone a gastrostomy (ALSFRS-R 5a) and walking (ALSFRS-R 8) being responsible for both types of differences. Misclassifications can be sources of bias for the predicted outcomes, so users of a CDSS that incorporates this model should be aware of those. A future study could work on enhancing the CDSS with awareness of what leads to a misclassification based on specific values, and leave the classification of certain instances to the domain expert. Additionally, although this study is a proof of concept, future data collection and evaluation can assess whether those errors are due to the subjective nature of QoL as an outcome, or other reasons such as outliers in the current dataset, or the small sample size.

### Towards the development of a clinical decision support system (CDSS)

It is important to first emphasise that the reason for the creation of CDSS is anthropocentric: they aim to improve healthcare which will, directly or indirectly, result in improving the lives of people who are affected by a disease. This idea is something we should have in mind throughout the entire process of conceiving to finally using and maintaining such a system. So, our aim is to create a system that will be safe and usable and will have a positive impact on healthcare.

In this study we worked towards creating a model to help clinicians to more easily assess the impact that ALS has on caregiver’s of PALS, as caregivers are of paramount importance to their care. Our aim was to incorporate this model in the future development of a CDSS, to automatically predict a caregiver’s QoL at the point of the patient’s care. The model we developed to this end used ten features that are routinely collected for the patient (such as the ALSFRS-R scores for the patient’s functionality assessment), can be a one-off collection (patient’s age of onset and employment status pre-symptoms, or caregiver’s age), or can be easily collected from the caregiver (hours of care, health). This way the clinician can be easily and quickly informed on the caregiver’s status too and suggest the necessary supports. The need for a small number of features does not only concern the usability of the system, but also the compliance with the European General Data Protection Regulations (GDPR, https://gdpr-info.eu/art-5-gdpr/). One of the GDPR principles is the data minimisation principle, according to which we ought to be collecting the least amount of data that are necessary for a task.

Another aspect that we aim to add to our model is explainability. The need for explainability has been supported by a popular interpretation of the GDPR Articles 13-15 (regarding “information to be provided where personal data are collected from the data subject”), and Article 22 (referring to a “right not to be subject to automated decision-making”), that claims the requirement for a “right to explanation” of the decisions that are made by automated systems, but this is a debatable statement. Whachter *et al*., 2017^39^ have argued that there is ambiguity in those Articles and what has been referred to as a requirement for a “right to explanation” by others, is not guaranteed by the GDPR and is merely a “right to be informed”. Regardless of the clarity and explicitness of the GDPR, it is important to create a system that is transparent, both for usability and for ethical purposes. One of the aspects of transparency is of course the explainability of a system, which has been found to have been included as a key feature in only a limited number of CDSS so far^40^. For this reason, we have incorporated a *post-hoc* explainability technique in our “black-box” model, which is supported by visual explanations to give a clearer picture of the decision-making process to the user. The transparency of a system is enhanced by the provision of information regarding the system’s correct or incorrect classifications to its future user. For this reason, we included not only the evaluation metrics but also an analysis of misclassifications in this study. Future users can be aware of when and why the CDSS makes an error and be in a position to better assess the suggestion.

Addressing all these aspects of transparency and data privacy can lead to better trust of the system by the people it concerns. Trust in the system and usability of it will eventually lead to its incorporation in clinical practice and better healthcare outcomes. The next step would be to evaluate the model on new participants and prospective users. The final model, as with most CDSS, should be subsequently tested in real clinical environments and it should be frequently re-validated as there can be changes in context as the time passes. This is the reason that some features such as financial information were not included in the CDSS models.

## Conclusion and future work

This study found new factors relevant to caregiver QoL in ALS, which included both patient and caregiver characteristics. The factor with the highest impact was the caregiver’s existential aspect of QoL. Due to the rarity of the condition, these findings are of great significance to the study of ALS; small studies can be combined to create a bigger picture of the disease.

Additionally, with the use of ML and XAI we created a model that acts as a proof of concept of the potential of AI to support the decision-making process in healthcare. Further work on the analysis of misclassifications and further evaluation of the current model can gradually lead to the creation of a CDSS that will be trusted and used by clinicians to accelerate the necessary actions for the support of both patient and caregiver. QoL is an outcome that is challenging to model as its assessment is subjective. Although we managed to achieve good predictive power, it is important to conduct more studies in order to understand whether the erroneous predictions are a result of the subjective experience of QoL or of the small sample size.

Future work is necessary for the evaluation of the models on new participants, both in Ireland but also on a broader population to test their generalisability. Additionally, as the development of a CDSS is a collaborative process, further consultancy with domain experts on the findings as well as user studies for the evaluation of the model in terms of usability are required.
